# The Distribution of HIV and AIDS Cases in Luzhou, China, From 2011 to 2020: Bayesian Spatiotemporal Analysis

**DOI:** 10.2196/37491

**Published:** 2022-06-14

**Authors:** Ningjun Ren, Yuansheng Li, Ruolan Wang, Wenxin Zhang, Run Chen, Ticheng Xiao, Hang Chen, Ailing Li, Song Fan

**Affiliations:** 1 School of Public Health Southwest Medical University Luzhou China; 2 Department of HIV/AIDS Control and Prevention Luzhou Center For Disease Control and Prevention Luzhou China

**Keywords:** HIV and AIDS, reported incidence, Bayesian model, spatio-temporal distribution

## Abstract

**Background:**

The vastly increasing number of reported HIV and AIDS cases in Luzhou, China, in recent years, coupled with the city’s unique geographical location at the intersection of 4 provinces, makes it particularly important to conduct a spatiotemporal analysis of HIV and AIDS cases.

**Objective:**

The aim of this study is to understand the spatiotemporal distribution of HIV and the factors influencing this distribution in Luzhou, China, from 2011 to 2020.

**Methods:**

Data on the incidence of HIV and AIDS in Luzhou from 2011 to 2020 were obtained from the AIDS Information Management System of the Luzhou Center for Disease Control and Prevention. ArcGIS was used to visualize the spatiotemporal distribution of HIV and AIDS cases. The Bayesian spatiotemporal model was used to investigate factors affecting the spatiotemporal distribution of HIV and AIDS, including the gross domestic product (GDP) per capita, urbanization rate, number of hospital beds, population density, and road mileage.

**Results:**

The reported incidence of HIV and AIDS rose from 8.50 cases per 100,000 population in 2011 to 49.25 cases per 100,000 population in 2020—an increase of 578.87%. In the first 5 years, hotspots were concentrated in Jiangyang district, Longmatan district, and Luxian county. After 2016, Luzhou’s high HIV incidence areas gradually shifted eastward, with Hejiang county having the highest average prevalence rate (41.68 cases per 100,000 population) from 2011 to 2020, being 2.28 times higher than that in Gulin county (18.30 cases per 100,000), where cold spots were concentrated. The risk for the incidence of HIV and AIDS was associated with the urbanization rate, population density, and GDP per capita. For every 1% increase in the urbanization rate, the relative risk (RR) increases by 1.3%, while an increase of 100 people per square kilometer would increase the RR by 8.7%; for every 1000 Yuan (US $148.12) increase in GDP per capita, the RR decreases by 1.5%.

**Conclusions:**

In Luzhou, current HIV and AIDS prevention and control efforts must be focused on the location of each district or county government; we suggest the region balance urban development and HIV and AIDS prevention. Moreover, more attention should be paid to economically disadvantaged areas.

## Introduction

HIV and AIDS have been prevalent in China for more than 30 years. HIV and AIDS have become one of China’s significant public health problems as it causes suffering to patients and seriously hinders healthy socioeconomic development [1]. Luzhou, located in southeastern Sichuan Province, is a central city in the combined region of Sichuan, Yunnan, Guizhou, and Chongqing, and is also an area in Sichuan Province, where the reported HIV epidemic is growing rapidly [2,3]. Despite considerable efforts by local governments, there remains much work required to fulfill the Political Declaration requirements on HIV and AIDS [4].

At present, domestic research on AIDS mainly focuses on epidemiology, prevention and control, clinical characteristics, etiology, and sociology [1]. By contrast, less research has been conducted on the process of its spatiotemporal spread. Nevertheless, some epidemiology studies have shown that the spread and distribution of HIV are closely related to geospatial factors [5]. Furthermore, although traditional regression models require variables of individuals to be independent of each other, these variables are likely to be correlated with each other owing to the influence of a familiar environment. Therefore, to identify deeper risk factors, researchers use Bayesian spatiotemporal models to be consistent with the correlation between individuals; to identify deeper risk factors, researchers use Bayesian spatiotemporal models that take into account spatial correlations. Yin et al [6] have used Bayesian spatiotemporal analysis to discover the impact of urbanization and residence on tuberculosis in other areas. Tian et al [7] have analyzed the impact of urbanization on the prevalence of scarlet fever. Card et al [8] reviewed the application of geographic information systems in HIV and emphasized the need for careful planning of resources concerning the geospatial movement and location of people living with HIV. Therefore, this study uses a Bayesian spatiotemporal model to analyze the impact of relevant data on the spatiotemporal distribution of HIV in Luzhou from 2011 to 2020 and provide a point of reference for the precise prevention and control of HIV in other prefecture-level cities in southwest China.

## Methods

### Ethics Approval

This study has been approved by the ethics committee of Southwest Medical University (KY2020225).

### Data Sources

The data on reported HIV and AIDS cases at district and township levels, in Luzhou, Sichuan province, from January 2011 to December 2020 were obtained from the AIDS Information Management System of the Luzhou Centre for Disease Control and Prevention; the date of registered onset and current address were collected for each case. Population data for each administrative district or township at year-end were collected from the Luzhou Bureau of Statistics, covering the years 2011 to 2020, obtained from the statistical yearbook [9] of the Luzhou Bureau of Statistics and relevant data provided by the Luzhou Health and Wellness Commission.

We downloaded the fundamental geographic data of municipal boundary with a scale of 1:400,000 from the National Geomatics of China, using ArcGIS (version 10.5; Environmental Systems Research Institute) to describe the spatial distribution of HIV and AIDS in Luzhou at the district and street levels. HIV and AIDS incidence rates were calculated at the city, district (county), and street (township) levels; a Bayesian spatiotemporal analysis was performed at the district and county levels, and spatial autocorrelation analysis was performed at the street and township levels.

### Analysis of Demographic Characteristics

We collected the following demographic information from the Luzhou Statistical Yearbook for use in the Bayesian spatiotemporal model and changed the units of some of the data to improve the final presentation: (1) population (the number of individuals who have lived in Luzhou for more than 6 months); (2) gross domestic product (GDP) per capita (the GDP divided by the population of the region), with 1000 Yuan (US $147.48) as the unit; (3) the urbanization rate (which is divided by the county’s resident population); (4) disposable income per capita (the sum of the final consumption expenditures and savings available to residents; ie, the income available for discretionary use), using 1000 Yuan (US $147.48) as the unit; (5) total road mileage in the territory (the length of roads within the districts and counties of Luzhou); (6) the number of practicing (assistant) physicians and hospital beds (the number of physicians and beds per 1000 people within the area during the observation period); (7) and population density (the number of permanent residents divided by the total area of the district or county). The above data are based on the yearbook published in the current year.

### Spatial Autocorrelation Analysis

Spatial autocorrelation statistics have been commonly used to understand the spatial distribution and structure of diseases; they also allow for examining spatial dependence or autocorrelation in spatial data [10,11]. According to Waldo Tobler’s first law of geography, “everything is related to everything else, but near things are more related than distant things”; therefore, neighboring counties’ or townships’ incidence rates of HIV should be more similar than those of nonneighboring counties or townships [12]. Spatial autocorrelation includes global spatial autocorrelation, which is used to estimate the overall degree of autocorrelation of spatial data, and local indicators of spatial association (LISA), which are used to assess the impact of individual locations on global statistics and determine the location and type of clusters. We performed all of the above analyses using GeoDa (version 1.10.0.8; Center for Spatial Data Science).

The Moran *I* is computed as follows [13]:

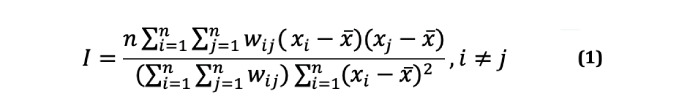



LISA are computed as follows [14]:

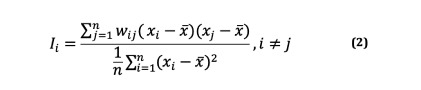



Where n is the number of districts, *x_i_* and *x_j_* are the values of the reported HIV and AIDS cases of districts *i* and *j*, respectively. *x̄* represents the average of all district-reported HIV and AIDS cases, and *w_ij_* is the spatial weight matrix corresponding to the district pair *i* and *j*. In calculating the global autocorrelation, the Moran *I*, a negative correlation is indicated when *I*＜0 and *P*＜.05, and a positive correlation is indicated when *I*＞0 and *P*＜.05; the larger the value of *I*, the more obvious the spatial correlation.

### Bayesian Spatiotemporal Model Analysis

We studied the impact of the resident population, urbanization rate, disposable income per capita, GDP per capita, road mileage, number of physicians, and population density on the reported incidence of HIV using HIV case data and population data for each district from 2011 to 2020. In this study, it was assumed that the number of HIV cases in the *i*th (*i*=1,2,…,7) district in the *t*th (*t*=1,2,…,10) year followed a Poisson distribution, meaning *y_it_* ˜ Poisson(λ*_it_*) and *E*(*y_it_*) = λ*_it_* = *e_it_θ_it_*.

*e_it_* denotes the expected number of HIV cases in year *t* in district *I*; *θ_it_* denotes the ratio of the number of actual cases to the expected number of cases in year *t* in district *i*, which is the RR of disease incidence. We use the log function form of *θ_it_* to build a Bayes model, computed as follows [15]. β_0_ is the intercept, *x_i_* (*i* =1,2, ..., 6) represents the urbanization rate, disposable income per capita, GDP per capita, road mileage, physicians, and density, respectively. β_1_ to β_6_ denote the regression coefficients of the corresponding variables.

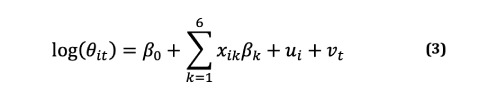



*u_i_* is the spatial structure effect, reflecting spatial dependence, which is assumed to obey a conditional autoregressive process, with a Gaussian distribution, and the mean being the weighted average of neighboring regions *u_j_*, *i* ≠ *j*, computed as follows [16], where *δ_i_* is the first-order neighborhood of region *i*, 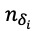 is the number of neighboring regions in region *i*, and *σ*^2^*_e_* is the variance of the spatial effect. *v_t_* is the temporal structure effect for which the prior distribution is a first-order autoregressive AR(1), where the temporal effect *v_t_* at time *t* is only related to the temporal effect *v_t_*_- 1_ at the previous time (ie, *v_t_* = *ρv_t_*_- 1_ + *Ɛ_t_*).

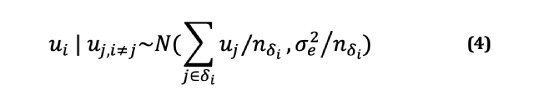



The Bayesian spatiotemporal model analysis applies the CARBayesST and CARBayes packages in R (version 4.1.0; R Foundation for Statistical Computing) to estimate parameter values using Markov chain Monte Carlo simulations, resulting in mean values and 95% CIs for the posterior estimates of the parameters [17].

## Results

### Spatiotemporal Analysis of HIV and AIDS Incidence

**Figure 1:**
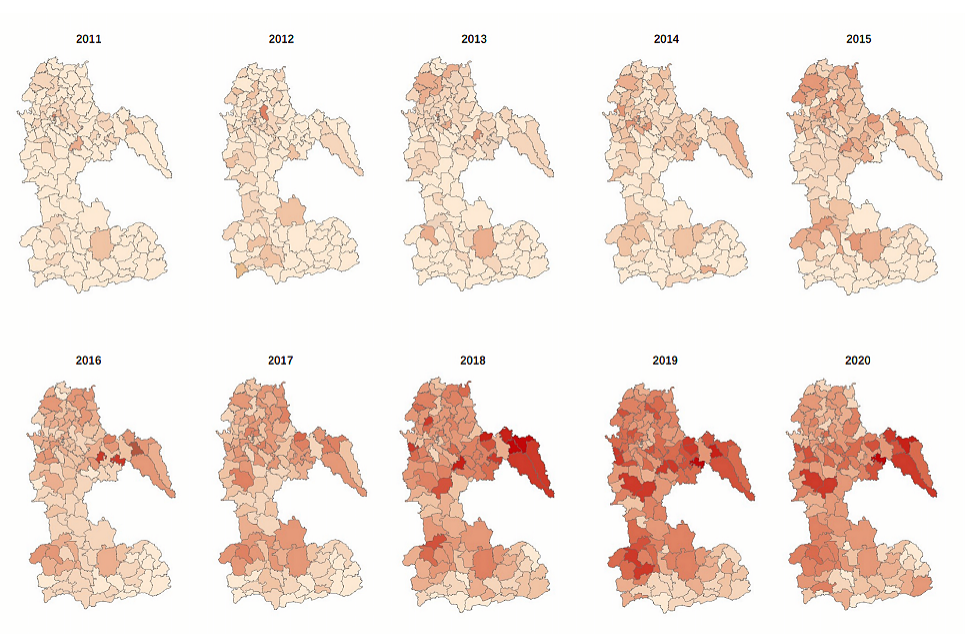
Reported HIV and AIDS incidence rates by street/township in Luzhou from 2011 to 2020.

A total of 13,111 HIV and AIDS cases were reported in Luzhou, Sichuan province, from 2011 to 2020. The reported incidence of HIV and AIDS rose from 8.50 cases per 100,000 population in 2011 to 49.25 cases per 100,000 in 2020, an increase of 578.87%. Table 1 shows that Hejiang county has the highest average incidence (41.68 cases per 100,000 populations) from 2011 to 2020, 2.28 times higher than that in Gulin county (18.30 cases per 100,000 population), which has the lowest average incidence rate. The highest cumulative number of cases occurred in Hejiang and Luxian counties, with 2904 and 2758 cases, respectively, during the last 10 years. In contrast, Gulin county has the lowest cumulative number of cases, only (1238/2904, 42.6%) of the cases in Hejiang county. The number of cases in each region showed an increasing trend year by year. The highest number of cases occurred in 2019, with 2850 new cases citywide, of which 688 cases were reported in Hejiang county. Figure 1 shows the change in HIV and AIDS in Luzhou from 2011-2020, where darker the color, more the HIV and AIDS cases.

**Table 1:** The number of HIV and AIDS cases and incidence rates by district and county in Luzhou from 2011 to 2020.

Year	Cases per district, n/N (per 100,000)						
	Jiangyang	Naxi	Longmatan	Luxian	Hejiang	Xuyong	Gulin
2011	59/585,000 (10.09)	30/445,000 (6.74)	72/352,000 (20.45)	71/832,000 (8.53)	59/707,000 (8.35)	29/581,000 (4.99)	38/706,000 (5.38)
2012	69/586,000 (11.77)	35/443,000 (7.90)	62/358,000 (17.32)	99/825,000 (12.00)	66/706,000 (9.35)	66/578,000 (11.42)	46/701,000 (6.56)
2013	95/595,000 (15.97)	48/427,000 (11.24)	69/360,000 (19.17)	185/824,000 (22.45)	94/697,000 (13.49)	69/572,000 (12.06)	79/692,000 (11.42)
2014	135/606,000 (22.28)	61/418,000 (14.59)	88/369,000 (23.85)	163/814,000 (20.02)	126/695,000 (18.13)	67/568,000 (11.80)	80/685,000 (11.68)
2015	136/526,000 (25.86)	72/408,000 (17.65)	115/384,000 (29.95)	278/816,000 (34.07)	154/699,600 (22.01)	109/566,000 (19.26)	93/678,000 (13.72)
2016	168/654,000 (25.69)	127/399,000 (31.83)	116/407,000 (28.50)	298/807,000 (36.93)	262/696,000 (37.64)	137/564,000 (24.29)	103/672,000 (15.33)
2017	235/681,000 (34.51)	197/389,000 (50.64)	145/426,000 (34.04)	309/797,000 (38.77)	276/695,000 (39.71)	171/562,000 (30.43)	139/664,000 (20.93)
2018	257/707,000 (36.35)	292/378,000 (77.25)	199/444,000 (44.82)	467/786,000 (59.41)	646/692,000 (93.35)	274/559,000 (49.02)	230/660,000 (34.85)
2019	394/737,000 (53.46)	351/367,000 (95.64)	216/464,000 (46.55)	565/773,000 (73.09)	688/691,000 (99.57)	406/556,000 (73.02)	230/656,000 (35.06)
2020	361/762,000 (47.38)	202/355,000 (56.90)	184/480,000 (38.33)	323/765,000 (42.22)	533/689,000 (77.36)	293/553,000 (52.98)	200/652,000 (30.67)
Average	1909/6,439,000 (29.65)	1415/4,029,000 (35.12)	1266/4,044,000 (31.31)	2758/8,039,000 (34.31)	2904/6,967,600 (41.68)	1621/5,659,000 (28.64)	1238/6,766,000 (18.30)

### Bayesian Analysis of HIV and AIDS Cases

**Figure 2:**
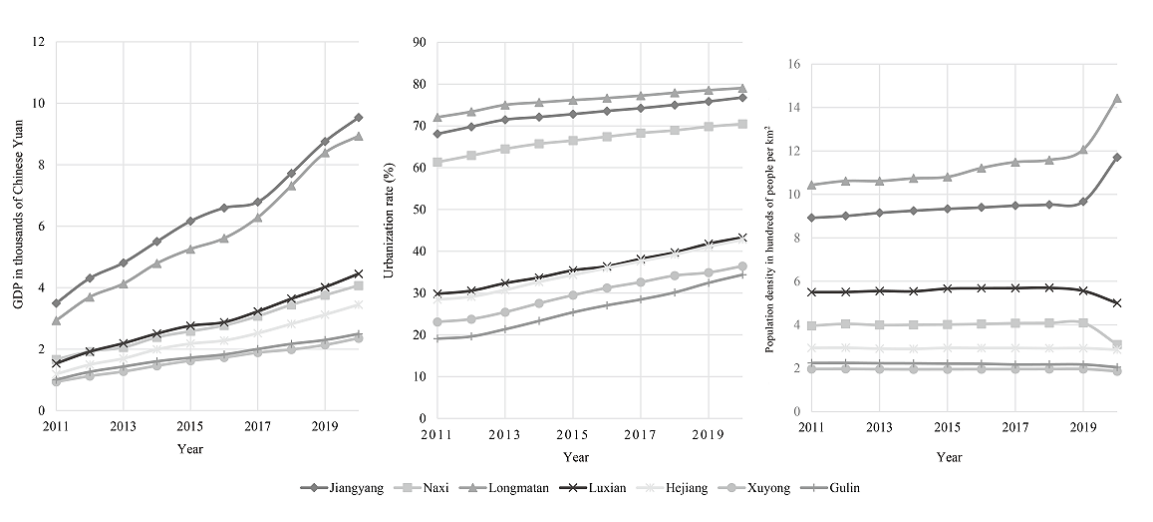
Trends in gross domestic product (GDP) per capita, population density, and urbanization rate by district and county.

We used a Bayesian spatiotemporal model to analyze factors such as urbanization rate, GDP per capita, road mileage, physicians, beds, and density, finding that urbanization rate and density increased the RR of having HIV, while GDP was a protective factor. For every 1% increase in the urbanization rate, the RR increases by 1.3%, while an increase of 100 people per square kilometer would increase the RR by 8.7%. Furthermore, for every 1000 Yuan (US $148.12) increase in GDP per capita, the RR value decreases by 1.5%. By contrast, the influence of the number of beds and road mileage on the risk of acquiring an HIV infection was not significant (Table 2). From 2011 to 2020, the GDP per capita of Luzhou City rose from 17,000 Yuan (US $2518.00) to 48,100 Yuan (US $7124.45), and the urbanization rate rose from 38.8% to 52%, while the population density remained at approximately 350 people per square kilometer, as shown in Figure 2.

**Table 2:** Bayesian model regression coefficient values.

Variable	Median (95% CI)	A posteriori estimated relative risk values (95% CI)
Gross domestic product per capita	–0.016 (–0.0296 to –0.0048)	0.985 (0.973 to 0.999)
Urbanization rate	0.014 (0.004 to 0.027)	1.013 (1.000 to 1.027)
Density	0.092 (0.033 to 0.159)	1.087 (1.020 to 1.164)
Road mileage	0.002 (0.000 to 0.005)	1.002 (1.000 to 1.004)
Number of beds	0.018 (–0.035 to 0.069)	1.019 (0.967 to 1.074)

### Spatial Autocorrelation Analysis of HIV and AIDS Incidence

The HIV and AIDS incidence in Luzhou at the township (street) level from 2011 to 2020 is shown in Multimedia Appendix 1. The highest incidence occurred in Danlin Township, Anfu Street, Fuji Township, Hetou Township, and Ganyu Township. Table 3 shows the results of the spatial autocorrelation analysis on HIV and AIDS incidence over the last decade, with Moran *I* values ranging from 0.174 to 0.483 (*P*<.05 for each Moran *I* value). This analysis indicates a high positive spatial autocorrelation of HIV and AIDS incidence at the street (township) level.

**Figure 3:**
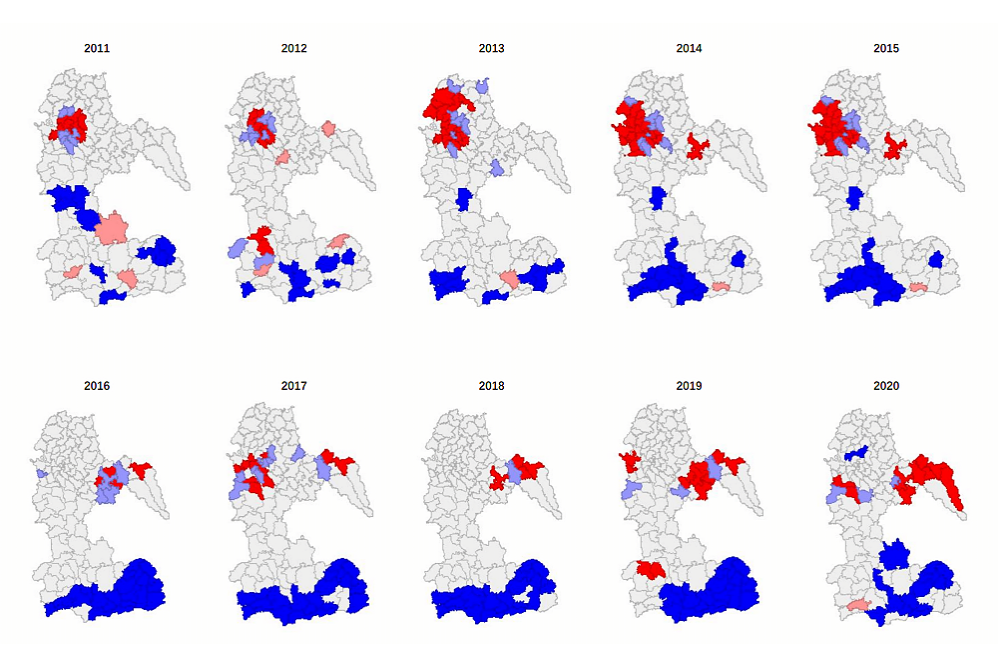
Local indicators of spatial association cluster map of HIV and AIDS incidence in Luzhou from 2011 to 2020.

Figure 3 illustrates the distribution of LISA values across Luzhou City. It shows the high-high (HH) incidence clusters in Taian township, Anfu township, and Lianhuachi township from 2011 to 2015. Since 2016, HH incidence clusters have moved toward the southeast, including some streets in Hejiang county. Most townships in Naxi county, Longmatan district, and Luxian county have transitioned to being without obvious spatial clustering after 2015. This analysis also showed that clusters of “cold spots” in core low-low areas were located in most townships in Gulin county. A map of the districts and counties of Luzhou is provided in Multimedia Appendix 2 to facilitate clarification of the administrative divisions of Luzhou.

**Table 3:** The results of the spatial autocorrelation test on HIV and AIDS incidence in China from 2011 to 2020.

Year	Moran *I*	*E(I)*	*Z* value	*P* value
2011	0.211	–0.0082	4.1506	.003
2012	0.174	–0.0082	3.3516	.005
2013	0.246	–0.0082	4.3966	.001
2014	0.308	–0.0082	5.5326	.001
2015	0.317	–0.0082	5.8649	.001
2016	0.336	–0.0082	6.0172	.001
2017	0.421	–0.0082	7.3177	.001
2018	0.483	–0.0082	8.3788	.001
2019	0.444	–0.0082	7.9508	.001
2020	0.411	–0.0082	7.0059	.001

## Discussion

This study analyzed the spatial and temporal distribution of HIV and AIDS incidence in Luzhou based on the number of HIV and AIDS cases and demographic data from 2011 to 2020 using Markov chain Monte Carlo methods and Bayesian spatiotemporal models. The Bayesian spatiotemporal model integrated the temporal information, spatial information, parameter uncertainty (prior distribution) embedded, and correlated factors associated spatiotemporally, which resolved the estimation bias caused by the spatial structure and made the estimates more stable and reliable [16]. We found that for every 1% increase in the urbanization rate, the RR value increases by 1.3%, while an increase of 100 people per square kilometer would increase the RR by 8.7%. In contrast, for every 1000 Yuan (US $148.42) increase in GDP per capita, the RR value decreases by 1.5%. Thus, density and urbanization rate may be essential factors in the rise in HIV and AIDS incidence in Luzhou, while GDP per capita slows its rise.

Previous studies based on Bayesian spatiotemporal models had shown that urbanization is positively associated with the prevalence of infectious diseases such as scarlet fever and tuberculosis [6,7]; it has also been shown that population density is associated with hemorrhagic fever in renal syndrome [18]. This study also found that increased urbanization and population density increased the RR of having HIV or AIDS. The urbanization rate increased by 4.88%, and population density increased by 6 persons per square kilometer (varying by county) from 2011 to 2020, suggesting that this may be one of the underlying reasons for the increase in reported HIV and AIDS incidence in Luzhou. By contrast, GDP per capita emerged as a protective factor against the incidence of HIV and AIDS. Consistent with previous studies, the regions with the worst AIDS epidemics globally were often less economically developed regions; sub-Saharan Africa, where 40% of the population lives below the poverty line of US $1.40 per day, had the highest incidence of AIDS globally [19].

Further analysis of our data for the past 10 years for the 7 counties of Luzhou showed that Longmatan district and Jiangyang district had the highest urbanization rates with the minor differences and the highest and fastest-growing GDP per capita; this area is also the economic, cultural, medical, and educational center of Luzhou with relatively complete infrastructure. However, its population density far exceeds that of other counties, increasing the RR. Combining the 3 factors, the reported HIV and AIDS incidence in the region is at a medium level. Therefore, it is suggested that the region balance urban development and HIV and AIDS prevention, increase publicity on high-risk behavior, raise awareness of self-protection methods, and increase investment in HIV and AIDS prevention and treatment.

The highest HIV and AIDS incidence was in Hejiang County, which increased to 91.74% in 2018 (compared to only 39% in 2017). This increase had implications for the predictions of the Bayesian spatiotemporal model. The GDP per capita, urbanization rate, and population density had minor variations around 2018, so we believe that the emergence of this phenomenon was related to Luzhou’s policies [20,21]. The policies increased screening for HIV and AIDS, and more cases are being detected as a result. This allowed some HIV and AIDS cases in patients who had been infected for a long time but were not aware of it to be detected earlier. Early detection is an integral part of HIV and AIDS prevention and treatment [22,23], which can reduce the risk of transmission, ensure the efficiency of antiviral treatment, and increase the lifespan of individuals with HIV or AIDS, suggesting the need for early screening.

By contrast, Gulin, which had the lowest average HIV and AIDS incidence rate, has always shown a low incidence overall as it has not been the worst affected area, has been relatively stable in all factors, and is far from economic and cultural centers. However, in recent years, the population density in Gulin Township has increased, and the number of cases and incidence rates have continued to increase. With the increasing openness of sexual attitudes and the frequent occurrence of nonmarital commercial sex, Gulin Town will become a priority area for HIV and AIDS prevention and treatment in Luzhou.

To further verify the previously mentioned influencing factors, we conducted a spatial clustering analysis of HIV and AIDS in Luzhou at the street or township scale. We found that HIV and AIDS incidence hotspots were concentrated near the streets where each district and county government is located (Anfu Street, Hongxing Street, Fuji township, Hejiang township, Xuyong township, and Gulin township). As economic and social progress continues [24,25], and urbanization rates increase (especially near each district and county government), the population is becoming more and more concentrated and densely populated. From 2011 to 2015, these areas, centered on Nancheng Street in Jiangyang district, Hongxing Street in Longmatan district, and Fuji Township in Luxian, have the highest incidence of HIV and AIDS. In the case of the townships of Lushan county, for example, the GDP per capita has increased from 15,300 Yuan (US $2266.57) per person in 2011 to 44,500 Yuan (US $6592.30) per person in 2020, almost 3 times what it was 10 years ago, an increase that has meant the townships of Lushan county are no longer the hotspots they once were, providing evidence to suggest that economic growth is a protective factor against HIV and AIDS [26,27].

After 2016, Luzhou’s high HIV and AIDS incidence area gradually shifted eastward, mainly concentrating in Xiantan, Nantan, Bailu, and Ganyu townships in Hejiang county. The area has a low level of economic development and insufficient human resources for health compared to other areas. The GDP per capita in the area is lower than the average in Luzhou, and the low level of economic development may be one of the reasons for the high prevalence of AIDS in the township [28,29]; the cold spots in Luzhou from 2011 to 2020 were mainly concentrated in Xuyong county and southern Gulin county. The results of the small-scale hotspot analysis also verified the influence of urbanization, population density, and GDP per capita on the spatial and temporal distribution of HIV and AIDS in Luzhou.

There are still some limitations in this study. First, population data are all from the Luzhou City Statistical Yearbook and are collected at the county level only; population data at the street and township levels are estimated using data from the sixth census in 2010 [30], and the incidence rates were not accurate for each year. Second, the indicators included in this paper are all macrocontrol statistics, but the causes affecting the incidence of AIDS are complex and varied, and it may not be possible to cover all the influencing factors. Lastly, the incidence of AIDS is reported late [31], and it is expected that the delay or lag between the number of reported HIV and AIDS infections and the exact number of HIV and AIDS infections will result in a difference in RR. Therefore, further studies are needed to collect more detailed data and conduct more in-depth studies.

In conclusion, from 2011 to 2020, the incidence and number of HIV and AIDS cases in all districts and counties of Luzhou have increased significantly, and the work of prevention and treatment still faces many challenges [32]. This study suggests that increasing urbanization rates and population density may be important reasons for the rise in reported HIV and AIDS incidence in Luzhou, while the growth in GDP per capita plays a protective role. This study has important implications for the precise prevention and control of HIV and AIDS in other prefecture-level cities in southwest China.

## Appendix

Multimedia Appendix 1 Heat map of reported HIV incidence rates by street/township in Luzhou, China, from 2011 to 2020.

Multimedia Appendix 2 The district and county level map of Luzhou, China.

## Abbreviations

GDP: gross domestic product
RR: relative risk
