# Supplementary figures and images for: The Distribution of HIV and AIDS Cases in Luzhou, China, From 2011 to 2020: Bayesian Spatiotemporal Analysis
Source: JMIR Public Health Surveill. 2022 Jun 14;8(6):e37491. doi: 10.2196/37491 (PMC9240955; doi:10.2196/37491)

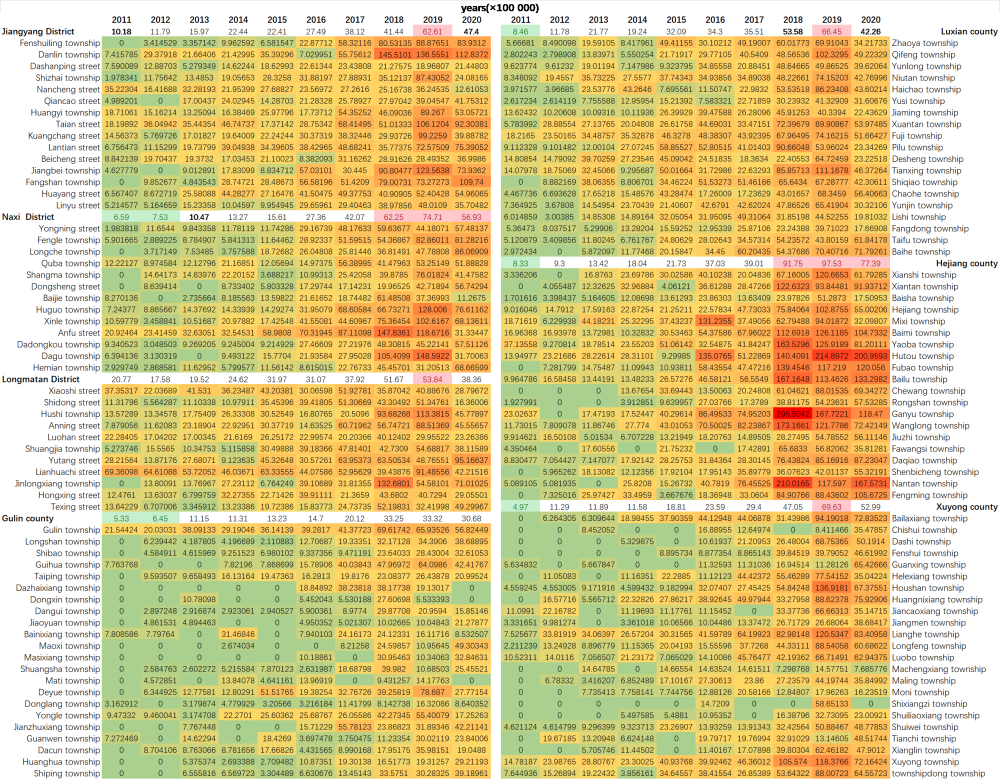

Supplement: Multimedia Appendix 1 [file publichealth_v8i6e37491_app1.png]

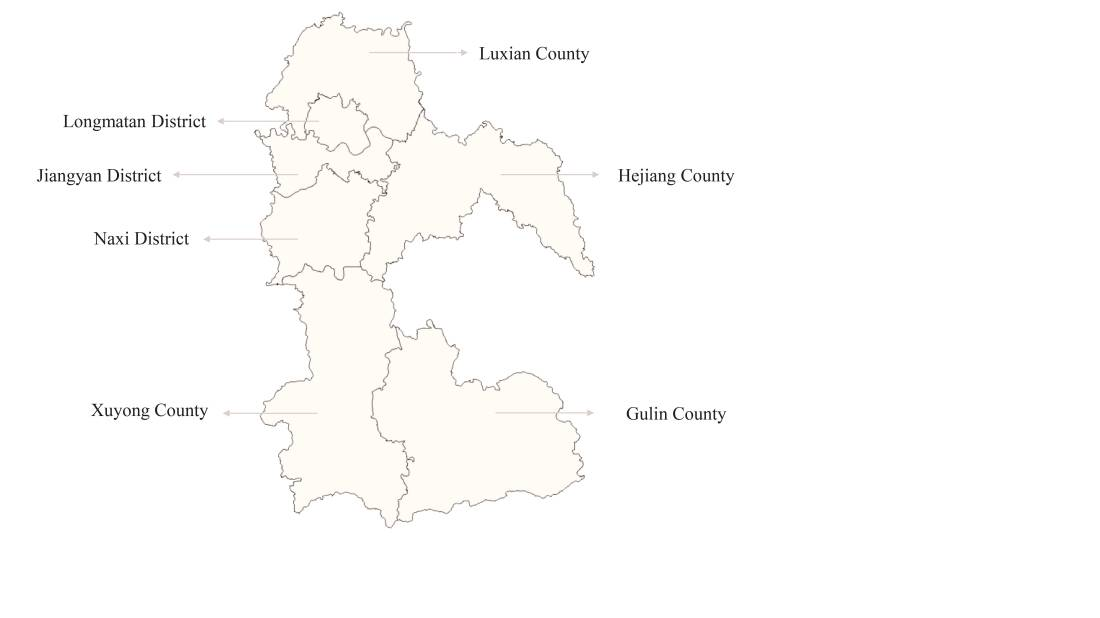

Supplement: Multimedia Appendix 2 [file publichealth_v8i6e37491_app2.png]
